# Poly[di-μ-glycinato-copper(II)]: a two-dimensional coordination polymer

**DOI:** 10.1107/S1600536811031503

**Published:** 2011-08-11

**Authors:** Fabienne Gschwind, Martin Jansen

**Affiliations:** a Max Planck Institute for Solid State Research, Heisenbergstrasse 1, 70569 Stuttgart, Germany

## Abstract

The title coordination polymer, [Cu(C_2_H_4_NO_2_)_2_]_*n*_, is two-dimensional and consists of a distorted octa­hedral copper coordination polyhedron with two bidentate glycine ligands chelating the metal through the O and N atoms in a *trans*-square-planar configuration. The two axial coordination sites are occupied by carbonyl O atoms of neighbouring glycine mol­ecules. The Cu—O distances for the axial O atoms [2.648 (2) and 2.837 (2) Å] are considerably longer than both the Cu—O [1.9475 (17) and 1.9483 (18) Å] and Cu—N [1.988 (2) and 1.948 (2) Å] distances in the equatorial plane, which indicates a strong Jahn–Teller effect. In the crystal, the two-dimensional networks are arranged parallel to (001) and are linked *via* N—H⋯O hydrogen bonds, forming a three-dimensional arrangement.

## Related literature

For the first work on cadmium glycinato complexes, see: Low *et al.* (1959 ▶). For similar mixed-metal glycinato complexes with copper(II), see: Papavinasam (1991 ▶); Davies *et al.* (2003 ▶); Low *et al.* (1959 ▶); Bi *et al.* (2006 ▶); Zhang *et al.* (2005 ▶). For further studies on cadmium–glycinato complexes, see: Barrie *et al.* (1993 ▶). For the properties and structure of a three-dimensional copper–glycinate polymer, see: Chen *et al.* (2009 ▶). For the synthesis of [NaCu_6_(gly)_3_(ClO_4_)_3_(H_2_O)]_*n*_(ClO_4_)_2*n*_, see: Aromi *et al.* (2008 ▶). 
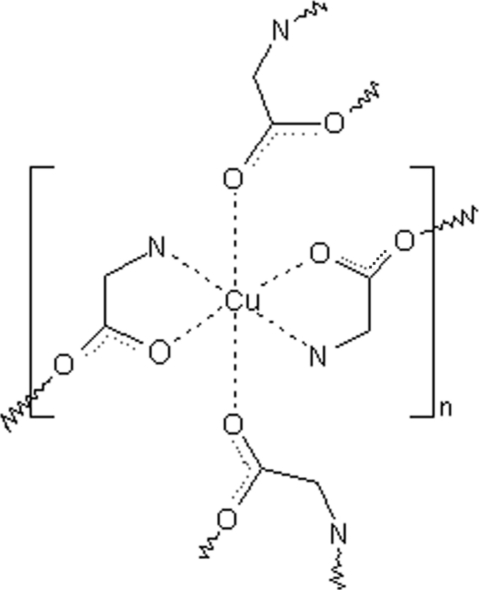


## Experimental

### 

#### Crystal data

[Cu(C_2_H_4_NO_2_)_2_]
*M* *_r_* = 211.66
Monoclinic, 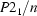

*a* = 9.4265 (19) Å
*b* = 5.1159 (10) Å
*c* = 13.912 (3) Å
β = 107.36 (3)°
*V* = 640.4 (2) Å^3^
*Z* = 4
Mo *K*α radiation
μ = 3.37 mm^−1^
*T* = 298 K
0.21 × 0.15 × 0.09 mm

#### Data collection

Stoe IPDS 2 diffractometer
Absorption correction: integration (*X-SHAPE* and *X-RED*; Stoe & Cie, 2009 ▶) *T*_min_ = 0.549, *T*_max_ = 0.692
9012 measured reflections
1876 independent reflections
1561 reflections with *I* > 2σ(*I*)
*R*_int_ = 0.048

#### Refinement

*R*[*F* ^2^ > 2σ(*F* ^2^)] = 0.032
*wR*(*F* ^2^) = 0.075
*S* = 1.03
1876 reflections
116 parameters
H atoms treated by a mixture of independent and constrained refinement
Δρ_max_ = 0.42 e Å^−3^
Δρ_min_ = −0.58 e Å^−3^

### 

Data collection: *X-AREA* (Stoe & Cie, 2009 ▶); cell refinement: *X-AREA*; data reduction: *X-RED* (Stoe & Cie, 2009 ▶); program(s) used to solve structure: *SHELXS97* (Sheldrick, 2008 ▶); program(s) used to refine structure: *SHELXL97* (Sheldrick, 2008 ▶); molecular graphics: *DIAMOND* (Brandenburg, 2006 ▶); software used to prepare material for publication: *SHELXL97*.

## Supplementary Material

Crystal structure: contains datablock(s) I, global. DOI: 10.1107/S1600536811031503/su2280sup1.cif

Structure factors: contains datablock(s) I. DOI: 10.1107/S1600536811031503/su2280Isup2.hkl

Additional supplementary materials:  crystallographic information; 3D view; checkCIF report

## Figures and Tables

**Table 1:** Hydrogen-bond geometry (Å, °)

*D*—H⋯*A*	*D*—H	H⋯*A*	*D*⋯*A*	*D*—H⋯*A*
N2—H1*A*⋯O3^i^	0.94 (5)	2.12 (5)	3.029 (3)	162 (4)
N2—H1*B*⋯O2^ii^	0.80 (4)	2.49 (4)	3.223 (3)	154 (4)
N1—H3*A*⋯O1^iii^	0.90 (4)	2.17 (4)	2.994 (3)	152 (3)
N1—H3*A*⋯O1^iv^	0.90 (4)	2.44 (4)	3.003 (3)	121 (3)
N1—H3*B*⋯O4^v^	0.86 (4)	2.41 (4)	3.152 (3)	145 (3)

Symmetry codes: (i) 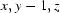; (ii) 
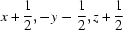
; (iii) 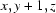; (iv) 
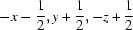
; (v) 
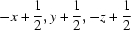
.

**Fig. 1.**
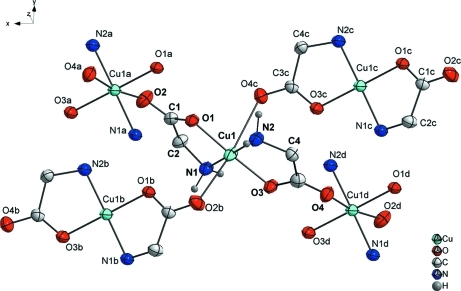
Part of the polymeric structure of the title compound, showing the numbering scheme and the displacement ellipsoids drawn at the 50% probability level [H atoms have been omitted for clarity; symmetry codes: (i) -x-1/2, y+1/2, -z+1/2; (ii) -x+1/2, y-1/2, -z+1/2].

**Fig. 2.**
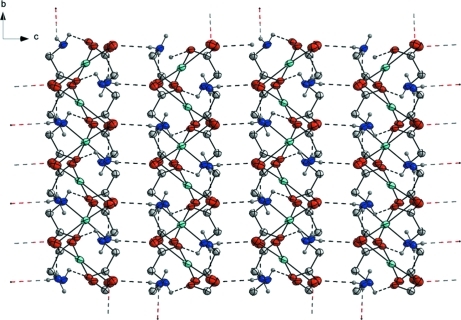
A view along the x-axis of the three-dimensional hydrogen bonded network of the title compound built up from the two-dimenional nets. The N-H···O hydrogen bonds are shown as dashed lines (see Table 1 for details; H-atoms not involved in these reactions have been omitted for clarity).

## supplementary crystallographic information

### Comment

Different metal glycine complexes and polymeric structures have been known since the 1960's. The first work on a cadmium glycinato complexe was done by (Low *et al.*, 1959), and further studies were reported by (Barrie *et al.*, 1993). Mixed metal glycinato complexes with copper(II) were investigated by (Papavinasam, 1991; Davies *et al.*, 2003; Low *et al.*, 1959).

The complexation of simple copper salts to amino acids is a well investigated reaction and various complexes and clusters have been reported (Low *et al.*, 1959; Davies *et al.*, 2003; Aromi *et al.*, 2008; Bi *et al.*, 2006; Zhang *et al.*, 2005). A three-dimensional copper-glycinate coordination polymer has been reported on by (Chen *et al.*, 2009).

While redissolving the copper cluster [NaCu_6_(gly)_3_(ClO_4_)_3_(H_2_O)]_n_ (ClO_4_)_2n_ (Aromi *et al.*, 2008) in DMSO, blue crystals of the title compound were obtained and were characterized by X-ray diffraction.

The title compound is a two-dimensional coordination polymer (Fig. 1). It consists of a distorted octahedral copper coordination polyhedron with two bidentate glycine ligands chelating the metal through the oxygen and nitrogen atoms (O1, O3, N1, N2) in a *trans* square planar configuration. The two axial coordination sites are occupied by carbonyl oxygen atoms of the neighbouring glycine molecules (O2 and O4). The Cu—O distances are 2.648 (2) Å (Cu1—O2^i^) and 2.837 (2) Å (Cu1—O4^ii^) for the axial oxygen atoms [symmetry codes: (i) -*x*-1/2, *y*+1/2, -*z*+1/2; (ii) -*x*+1/2, *y*-1/2, -*z*+1/2]. In the equatorial plane the Cu-O distances are 1.9474 (15) and 1.9483 (16) Å for Cu1—O1 and Cu1—O3, respectively, while the Cu—N distances are 1.9883 (19) and 1.948 (2) Å for Cu1-N1 and Cu1—N2, respectively. These bond length differences indicate a strong Jahn-Teller effect.

In the crystal the two dimensional networks are linked via N-H···O hydrogen bonds to form a three-dimensional arrangement (Table 1 and Fig. 2).

### Experimental

The title compound was prepared by dissolving 20 mg of [NaCu_6_(gly)_3_(ClO_4_)_3_(H_2_O)]_n_ (ClO_4_)_2n_ (Aromi *et al.*, 2008) in 5 ml DMSO. Crystals could be grown out of the blue solution by slow diffusion of THF.

### Refinement

The NH-atoms were located in difference electron-density maps and were freely refined. The C-bound H-atoms were included in calculated positions and treated as riding atoms: C-H = 0.97 Å, with U_iso_(H) = 1.2U_eq_(C).

### Crystal data

**Table:**

[Cu(C_2_H_4_NO_2_)_2_]	*F*(000) = 428
*M**_r_* = 211.66	*D*_x_ = 2.195 Mg m^−^^3^
Monoclinic, *P*2_1_/*n*	Mo *K*α radiation, λ = 0.71073 Å
Hall symbol: -P 2yn	Cell parameters from 5867 reflections
*a* = 9.4265 (19) Å	θ = 2.3–30.5°
*b* = 5.1159 (10) Å	µ = 3.37 mm^−^^1^
*c* = 13.912 (3) Å	*T* = 298 K
β = 107.36 (3)°	Block, blue
*V* = 640.4 (2) Å^3^	0.21 × 0.15 × 0.09 mm
*Z* = 4	

### Data collection

**Table:**

Stoe IPDS 2 diffractometer	1876 independent reflections
Radiation source: fine-focus sealed tube	1561 reflections with *I* > 2σ(*I*)
graphite	*R*_int_ = 0.048
Detector resolution: 6.67 pixels mm^-1^	θ_max_ = 30.0°, θ_min_ = 2.3°
rotation method scans	*h* = −13→13
Absorption correction: integration (*X-SHAPE* and *X-RED*; Stoe & Cie, 2009)	*k* = −7→6
*T*_min_ = 0.549, *T*_max_ = 0.692	*l* = −19→17
9012 measured reflections	

### Refinement

**Table:**

Refinement on *F*^2^	Primary atom site location: structure-invariant direct methods
Least-squares matrix: full	Secondary atom site location: difference Fourier map
*R*[*F*^2^ > 2σ(*F*^2^)] = 0.032	Hydrogen site location: inferred from neighbouring sites
*wR*(*F*^2^) = 0.075	H atoms treated by a mixture of independent and constrained refinement
*S* = 1.03	*w* = 1/[σ^2^(*F*_o_^2^) + (0.0447*P*)^2^] where *P* = (*F*_o_^2^ + 2*F*_c_^2^)/3
1876 reflections	(Δ/σ)_max_ = 0.001
116 parameters	Δρ_max_ = 0.42 e Å^−^^3^
0 restraints	Δρ_min_ = −0.58 e Å^−^^3^

### Special details

**Table:**

Geometry. Bond distances, angles etc. have been calculated using the rounded fractional coordinates. All su's are estimated from the variances of the (full) variance-covariance matrix. The cell esds are taken into account in the estimation of distances, angles and torsion angles
Refinement. Refinement of *F*^2^ against ALL reflections. The weighted *R*-factor *wR* and goodness of fit *S* are based on *F*^2^, conventional *R*-factors *R* are based on *F*, with *F* set to zero for negative *F*^2^. The threshold expression of *F*^2^ > σ(*F*^2^) is used only for calculating *R*-factors(gt) *etc*. and is not relevant to the choice of reflections for refinement. *R*-factors based on *F*^2^ are statistically about twice as large as those based on *F*, and *R*- factors based on ALL data will be even larger.

### Fractional atomic coordinates and isotropic or equivalent isotropic displacement parameters (Å^2^)

**Table:**

	*x*	*y*	*z*	*U*_iso_*/*U*_eq_	
Cu1	−0.00228 (3)	0.02678 (5)	0.26465 (2)	0.0301 (1)	
O1	−0.17587 (17)	−0.1989 (3)	0.21922 (12)	0.0270 (4)	
O2	−0.3924 (2)	−0.2410 (4)	0.10081 (14)	0.0408 (6)	
O3	0.17471 (18)	0.2461 (3)	0.30307 (13)	0.0317 (4)	
O4	0.41730 (18)	0.2283 (4)	0.38098 (15)	0.0392 (5)	
N1	−0.1151 (2)	0.2642 (4)	0.15535 (16)	0.0283 (5)	
N2	0.1137 (2)	−0.2140 (4)	0.37098 (17)	0.0302 (6)	
C1	−0.2742 (2)	−0.1247 (4)	0.13882 (17)	0.0260 (6)	
C2	−0.2384 (3)	0.1181 (4)	0.08778 (17)	0.0304 (6)	
C3	0.2916 (2)	0.1351 (4)	0.36051 (16)	0.0253 (5)	
C4	0.2694 (2)	−0.1268 (4)	0.40529 (17)	0.0282 (6)	
H1A	0.112 (5)	−0.378 (10)	0.340 (3)	0.076 (13)*	
H1B	0.082 (4)	−0.233 (7)	0.418 (3)	0.045 (9)*	
H2A	−0.21240	0.06770	0.02790	0.0360*	
H2B	−0.32560	0.22930	0.06700	0.0360*	
H3A	−0.153 (4)	0.393 (7)	0.184 (2)	0.045 (9)*	
H3B	−0.061 (4)	0.342 (8)	0.124 (3)	0.061 (11)*	
H4A	0.33140	−0.25670	0.38670	0.0340*	
H4B	0.30110	−0.11300	0.47810	0.0340*	

### Atomic displacement parameters (Å^2^)

**Table:**

	*U*^11^	*U*^22^	*U*^33^	*U*^12^	*U*^13^	*U*^23^
Cu1	0.0249 (1)	0.0214 (1)	0.0380 (2)	−0.0026 (1)	−0.0001 (1)	0.0078 (1)
O1	0.0262 (7)	0.0191 (7)	0.0334 (8)	−0.0016 (5)	0.0052 (6)	0.0030 (6)
O2	0.0375 (9)	0.0391 (10)	0.0389 (10)	−0.0142 (7)	0.0010 (7)	0.0043 (7)
O3	0.0277 (7)	0.0216 (7)	0.0408 (9)	−0.0028 (6)	0.0028 (6)	0.0046 (6)
O4	0.0275 (8)	0.0350 (9)	0.0510 (11)	−0.0060 (7)	0.0057 (7)	0.0016 (8)
N1	0.0274 (9)	0.0214 (8)	0.0340 (10)	−0.0019 (7)	0.0062 (7)	0.0055 (7)
N2	0.0291 (9)	0.0253 (9)	0.0331 (11)	−0.0023 (7)	0.0046 (8)	0.0068 (8)
C1	0.0282 (10)	0.0234 (9)	0.0262 (10)	−0.0011 (7)	0.0080 (8)	−0.0020 (8)
C2	0.0351 (11)	0.0263 (10)	0.0264 (11)	−0.0053 (8)	0.0041 (8)	0.0017 (8)
C3	0.0267 (9)	0.0238 (9)	0.0246 (10)	−0.0018 (7)	0.0066 (8)	−0.0025 (7)
C4	0.0279 (10)	0.0272 (10)	0.0272 (11)	0.0017 (8)	0.0049 (8)	0.0043 (8)

### Geometric parameters (Å, °)

**Table:**

Cu1—O1	1.9475 (17)	N2—C4	1.471 (3)
Cu1—O3	1.9483 (18)	N1—H3B	0.86 (4)
Cu1—N1	1.988 (2)	N1—H3A	0.90 (4)
Cu1—N2	1.984 (2)	N2—H1A	0.94 (5)
Cu1—O2^i^	2.648 (2)	N2—H1B	0.80 (4)
Cu1—O4^ii^	2.837 (2)	C1—C2	1.518 (3)
O1—C1	1.279 (3)	C3—C4	1.518 (3)
O2—C1	1.234 (3)	C2—H2A	0.9700
O3—C3	1.284 (3)	C2—H2B	0.9700
O4—C3	1.229 (3)	C4—H4A	0.9700
N1—C2	1.463 (3)	C4—H4B	0.9700
			
O1—Cu1—O3	176.59 (8)	H3A—N1—H3B	105 (4)
O1—Cu1—N1	84.73 (8)	C4—N2—H1A	107 (3)
O1—Cu1—N2	95.55 (8)	Cu1—N2—H1A	106 (3)
O1—Cu1—O2^i^	92.26 (7)	Cu1—N2—H1B	115 (3)
O1—Cu1—O4^ii^	80.68 (7)	C4—N2—H1B	111 (3)
O3—Cu1—N1	94.41 (8)	H1A—N2—H1B	108 (4)
O3—Cu1—N2	85.22 (8)	O2—C1—C2	119.5 (2)
O2^i^—Cu1—O3	91.07 (7)	O1—C1—O2	123.9 (2)
O3—Cu1—O4^ii^	96.01 (7)	O1—C1—C2	116.60 (19)
N1—Cu1—N2	178.27 (9)	N1—C2—C1	111.24 (19)
O2^i^—Cu1—N1	92.22 (8)	O3—C3—O4	124.2 (2)
O4^ii^—Cu1—N1	89.04 (8)	O3—C3—C4	116.60 (18)
O2^i^—Cu1—N2	89.48 (8)	O4—C3—C4	119.3 (2)
O4^ii^—Cu1—N2	89.32 (8)	N2—C4—C3	112.39 (18)
O2^i^—Cu1—O4^ii^	172.69 (7)	N1—C2—H2A	109.00
Cu1—O1—C1	115.30 (14)	N1—C2—H2B	109.00
Cu1^iii^—O2—C1	113.23 (15)	C1—C2—H2A	109.00
Cu1—O3—C3	114.93 (14)	C1—C2—H2B	109.00
Cu1^iv^—O4—C3	120.10 (16)	H2A—C2—H2B	108.00
Cu1—N1—C2	108.68 (14)	N2—C4—H4A	109.00
Cu1—N2—C4	109.16 (15)	N2—C4—H4B	109.00
C2—N1—H3A	108 (2)	C3—C4—H4A	109.00
Cu1—N1—H3A	107.6 (18)	C3—C4—H4B	109.00
Cu1—N1—H3B	114 (3)	H4A—C4—H4B	108.00
C2—N1—H3B	113 (3)		
			
N1—Cu1—O1—C1	6.99 (16)	N2—Cu1—O2^i^—C1^i^	−157.43 (17)
N2—Cu1—O1—C1	−171.29 (16)	O1—Cu1—O4^ii^—C3^ii^	−133.24 (18)
O2^i^—Cu1—O1—C1	99.01 (15)	O3—Cu1—O4^ii^—C3^ii^	47.61 (18)
O4^ii^—Cu1—O1—C1	−82.90 (15)	N1—Cu1—O4^ii^—C3^ii^	141.95 (18)
N1—Cu1—O3—C3	−166.00 (16)	N2—Cu1—O4^ii^—C3^ii^	−37.51 (18)
N2—Cu1—O3—C3	12.30 (16)	Cu1—O1—C1—O2	−178.31 (18)
O2^i^—Cu1—O3—C3	101.69 (16)	Cu1—O1—C1—C2	3.1 (2)
O4^ii^—Cu1—O3—C3	−76.51 (16)	Cu1^iii^—O2—C1—O1	32.3 (3)
O1—Cu1—N1—C2	−14.98 (16)	Cu1^iii^—O2—C1—C2	−149.11 (17)
O3—Cu1—N1—C2	161.71 (16)	Cu1—O3—C3—O4	169.39 (19)
O2^i^—Cu1—N1—C2	−107.04 (16)	Cu1—O3—C3—C4	−11.0 (2)
O4^ii^—Cu1—N1—C2	65.75 (16)	Cu1^iv^—O4—C3—O3	−34.4 (3)
O1—Cu1—N2—C4	166.43 (15)	Cu1^iv^—O4—C3—C4	146.03 (16)
O3—Cu1—N2—C4	−10.23 (15)	Cu1—N1—C2—C1	19.8 (2)
O2^i^—Cu1—N2—C4	−101.35 (15)	Cu1—N2—C4—C3	7.4 (2)
O4^ii^—Cu1—N2—C4	85.86 (15)	O1—C1—C2—N1	−15.8 (3)
O1—Cu1—O2^i^—C1^i^	−61.90 (17)	O2—C1—C2—N1	165.5 (2)
O3—Cu1—O2^i^—C1^i^	117.36 (17)	O3—C3—C4—N2	2.1 (3)
N1—Cu1—O2^i^—C1^i^	22.91 (17)	O4—C3—C4—N2	−178.3 (2)

Symmetry codes: (i) −*x*−1/2, *y*+1/2, −*z*+1/2; (ii) −*x*+1/2, *y*−1/2, −*z*+1/2; (iii) −*x*−1/2, *y*−1/2, −*z*+1/2; (iv) −*x*+1/2, *y*+1/2, −*z*+1/2.

### Hydrogen-bond geometry (Å, °)

**Table:**

*D*—H···*A*	*D*—H	H···*A*	*D*···*A*	*D*—H···*A*
N2—H1A···O3^v^	0.94 (5)	2.12 (5)	3.029 (3)	162 (4)
N2—H1B···O2^vi^	0.80 (4)	2.49 (4)	3.223 (3)	154 (4)
N1—H3A···O1^vii^	0.90 (4)	2.17 (4)	2.994 (3)	152 (3)
N1—H3A···O1^i^	0.90 (4)	2.44 (4)	3.003 (3)	121 (3)
N1—H3B···O4^iv^	0.86 (4)	2.41 (4)	3.152 (3)	145 (3)

Symmetry codes: (v) *x*, *y*−1, *z*; (vi) *x*+1/2, −*y*−1/2, *z*+1/2; (vii) *x*, *y*+1, *z*; (i) −*x*−1/2, *y*+1/2, −*z*+1/2; (iv) −*x*+1/2, *y*+1/2, −*z*+1/2.
